# Modeling human epigenetic disorders in mice: Beckwith-Wiedemann syndrome and Silver-Russell syndrome

**DOI:** 10.1242/dmm.044123

**Published:** 2020-05-26

**Authors:** Suhee Chang, Marisa S. Bartolomei

**Affiliations:** Epigenetics Institute, Department of Cell and Developmental Biology, Perelman School of Medicine, University of Pennsylvania, Philadelphia, PA 19104, USA

**Keywords:** Imprinting disorders, Beckwith-Wiedemann syndrome, Silver-Russell syndrome, *H19*, *Igf2*, *Cdkn1c*, *Kcnq1ot1*, Imprinting control regions

## Abstract

Genomic imprinting, a phenomenon in which the two parental alleles are regulated differently, is observed in mammals, marsupials and a few other species, including seed-bearing plants. Dysregulation of genomic imprinting can cause developmental disorders such as Beckwith-Wiedemann syndrome (BWS) and Silver-Russell syndrome (SRS). In this Review, we discuss (1) how various (epi)genetic lesions lead to the dysregulation of clinically relevant imprinted loci, and (2) how such perturbations may contribute to the developmental defects in BWS and SRS. Given that the regulatory mechanisms of most imprinted clusters are well conserved between mice and humans, numerous mouse models of BWS and SRS have been generated. These mouse models are key to understanding how mutations at imprinted loci result in pathological phenotypes in humans, although there are some limitations. This Review focuses on how the biological findings obtained from innovative mouse models explain the clinical features of BWS and SRS.

## Abstract (precis)

**Summary:** Beckwith-Wiedemann syndrome (BWS) and Silver-Russell syndrome (SRS) can be caused by various (epi)genetic lesions leading to the dysregulation of genomic imprinting. This Review focuses on the mouse models used to understand how such perturbations contribute to the human BWS/SRS phenotypes.

## Introduction

A small number of mammalian genes are expressed in a parent-of-origin-specific manner via an epigenetic phenomenon known as genomic imprinting (Barlow and Bartolomei, 2014). Imprinted genes are typically found clustered in the genome and have important developmental roles, including the control of fetal growth and placental function (Plasschaert and Bartolomei, 2014). Genomic imprinting is largely regulated by the differential DNA methylation of discrete genomic elements located within imprinted clusters, which are called imprinting control regions (ICRs) (Li et al., 1993; Edwards and Ferguson-Smith, 2007). ICRs are methylated on a single parental allele during gametogenesis. Normally, this methylation state is maintained throughout development, despite the genome-wide epigenetic reprogramming that occurs after fertilization in the early embryo (Barlow and Bartolomei, 2014). Offspring with improper DNA methylation, inherited or somatic mutations and partial deletions of ICRs may exhibit abnormal genomic imprinting. Perturbations of imprinted genes and their regulation often lead to abnormal pre- and postnatal growth, as exemplified by the human imprinting disorders Beckwith-Wiedemann syndrome (BWS), Silver-Russell syndrome (SRS), Prader-Willi syndrome and Angelman syndrome. In this Review, we discuss how mouse models have contributed to our understanding of two of these imprinting disorders: BWS and SRS.

## Why the mouse model?

Since the discovery of genomic imprinting (McGrath and Solter, 1984; Surani et al., 1984), mouse models have been a powerful tool to study genetic disorders associated with abnormal imprints. The main advantage of the mouse lies in the largely evolutionarily conserved mechanisms that govern genomic imprinting (Edwards and Ferguson-Smith, 2007; Lee and Bartolomei, 2013). For each imprinted cluster, the genes, ICRs and epigenetic modifications responsible for parent-of-origin-specific expression are largely conserved between mice and humans. Additionally, the prevalence of single-nucleotide polymorphisms in numerous mouse strains allows for elegant mating strategies, and tracking of the maternal and paternal alleles in offspring. Furthermore, the marks that designate the parental identity of imprinted genes are established in the germline and can readily be evaluated in murine gametes. Importantly, loss of imprinting often occurs early in development in cells and tissues that are not easily accessible in humans but are accessible in the mouse. Nearly 40 years after its discovery, mouse models continue to serve as a vital tool to understand the physiological consequences of genomic imprinting.

## BWS

BWS [Online Mendelian Inheritance in Man (OMIM) catalog #130650] is one of the most common fetal overgrowth syndromes, with an incidence of 1 in 10,000 to 13,700 births (Vora and Bianchi, 2009; Mussa et al., 2013). Clinical features include pre- and postnatal overgrowth, hemihypertrophy, macroglossia, neonatal hypoglycemia, hyperinsulinism and hypoglycemia, organomegaly, renal abnormalities, anterior abdominal wall defects, umbilical hernia, ear creases, increased susceptibility to congenital/childhood tumors such as Wilms' tumor, adrenocortical carcinoma, hepatoblastoma and neuroblastoma, placentomegaly and placental mesenchymal dysplasia (DeBaun et al., 2000; Bliek, 2001; Weksberg et al., 2010; Brioude et al., 2018a,b). Notably, these symptoms can vary substantially among individuals. Although the molecular etiology of BWS is multifold, the vast majority of cases involve genetic and epigenetic perturbations of two clusters of imprinted genes on chromosome 11p15: the *H19*/*IGF2* cluster and *CDKN1C*/*KCNQ1OT1* cluster.

## Imprinting mechanisms of the *H19/IGF2* cluster and *CDKN1C/KCNQ1OT1* cluster

**Fig. 1.**
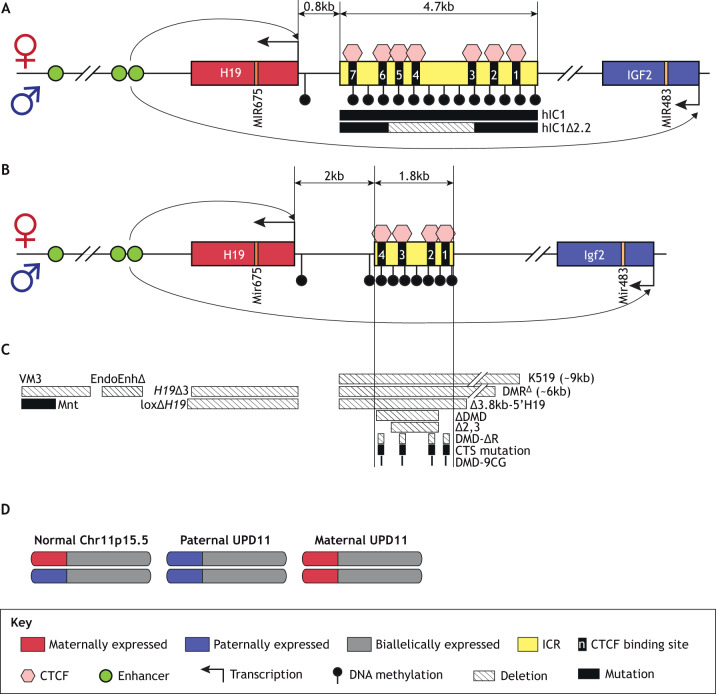
***H19*/*IGF2* imprinted locus.** (A) Human *H19*/*IGF2* cluster. On the maternal allele, CTCF binds to the unmethylated IC1 and insulates the *IGF2* promoter from the downstream enhancers, enabling *H19* expression. The methylated paternal IC1 silences the paternal *H19* allele and allows *IGF2* expression. *IGF2* is expressed from multiple promoters. MIR675 is located within the first exon of *H19*, whereas MIR483 is encoded within the second-to-last intron of *IGF2.* The BWS-related mutation (hIC1Δ2.2), which was introduced in the humanized IC1 allele in mice, is shown at the bottom of the panel. (B) Mouse *H19*/*Igf2* cluster. The *H19/Igf2* ICR is smaller in size and has fewer CTCF binding sites compared to human IC1, but the imprinting mechanism, detailed in A, is conserved between two species. (C) Summary of the mouse models described in this Review. Striped boxes indicate deletions and solid-filled boxes indicate mutations. (D) Simplified depiction of uniparental disomy (UPD). Sizes in kb are approximate and figures are not drawn to scale.

The *H19*/*IGF2* locus includes two imprinted genes located at chromosome 11p15.5 in human and at distal chromosome 7 in mouse (Fig. 1). In humans, telomeric to the ICR (designated as IC1) resides *H19*, which encodes a cytoplasmic long noncoding RNA (lncRNA). *H19* is associated with growth suppression, delayed placental growth, cell cycle regulation, cardiac remodeling, tumorigenesis and metastasis (Yoshimizu et al., 2008; Lecerf et al., 2019; Zhou et al., 2019). *H19* lncRNA may be processed into a microRNA (MIR675/Mir675), although its exact role remains unclear (Mineno et al., 2006; Keniry et al., 2012).

Centromeric to IC1 resides *IGF2*, which encodes insulin-like growth factor 2, a fetal growth-promoting protein essential for proper organ development, and an intronic microRNA (MIR483/miR483) (Fig. 1). In various tissues, IGF2 promotes cell proliferation and differentiation (Borensztein et al., 2013; Ferrón et al., 2015). In both mouse and human, *H19* and *Igf2* are regulated by the ICR and share tissue-specific enhancers located downstream of *H19* (Fig. 1A,B) (Leighton et al., 1995; Kaffer et al., 2001). The maternal ICR is unmethylated and is bound by the architectural protein CTCF (Bell et al., 1999; Bell and Felsenfeld, 2000; Lee and Bartolomei, 2013). CTCF maintains the unmethylated state of the maternal ICR and functions as an insulator to prevent the downstream enhancers from interacting with the maternal *Igf2* promoter (Yoon et al., 2007). Consequently, *Igf2* is silenced and *H19* is expressed on the maternal allele. Conversely, the paternal *H19*/*Igf2* ICR is methylated during spermatogenesis. Following fertilization, this methylation spreads to the paternal *H19* promoter and silences the paternal *H19* (Thorvaldsen et al., 1998). Additionally, the methylation of the paternal ICR interferes with CTCF binding and formation of the insulator. As a result, *Igf2* is expressed and *H19* is silenced on the paternal allele. The ICR is critical for allele-specific regulation of *Igf2* and *H19*, as shown in a mouse model with the ICR deletion [Fig. 1C, Δ3.8kb-5′H19 (Thorvaldsen et al., 2006) and K519 (Kaffer et al., 2000)]. When the *H19/Igf2* ICR was deleted on the maternal allele, the normally silent maternal *Igf2* was activated, increasing the fetal weight.

**Fig. 2.**
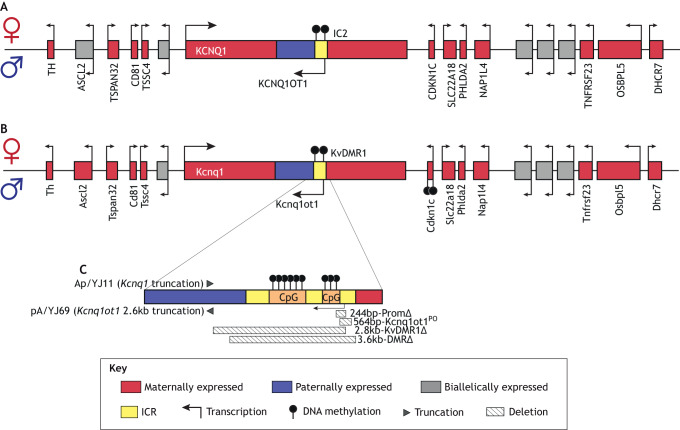
***CDKN1C*/*KCNQ1OT1* imprinted locus.** (A) Human *CDKN1C*/*KCNQ1OT1* cluster. On the maternal allele, IC2, which includes the transcription start site of *KCNQ1OT1*, is methylated. Thus, the maternal *KCNQ1OT1* is repressed, allowing the expression of maternal-allele-specific linked genes such as *KCNQ1* and *CDKN1C.* On the paternal allele, IC2 is unmethylated and *KCNQ1OT1* is transcribed, silencing the linked imprinted genes on the paternal allele. (B) Mouse *Cdkn1c*/*Kcnq1ot1* cluster. Note that IC2 is designated as KvDMR1 in mouse. The methylation on the *Cdkn1c* promoter and gene body is acquired postfertilization, and is absent in human (Bhogal et al., 2004). (C) Mouse models described in this Review. Striped boxes indicate deletions and filled triangles indicate insertions of truncation cassettes. Figures are not drawn to scale.

Centromeric to the human *H19*/*IGF2* cluster (Fig. 2A) and telomeric to the mouse cluster (Fig. 2B) is the *CDKN1C*/*KCNQ1OT1* cluster. The ICR of this cluster (designated as IC2 in humans and as KvDMR1 in mice) and its regulatory mechanisms are conserved. KvDMR1 regulates allelic expression of multiple imprinted genes in the cluster, including *Kcnq1ot1*, cyclin-dependent kinase inhibitor (*Cdkn1c* or *p57^Kip2^*), *Slc22a18*, *Phlda2*, *Ascl2* and *Kcnq1*. Each of these imprinted genes plays important roles in development. For example, *Cdkn1c* regulates cell proliferation and promotes cell cycle arrest (Hatada and Mukai, 1995; Matsuoka et al., 1996); *Kcnq1* encodes a potassium channel and is imprinted at specific developmental stages and tissues (Wang et al., 1996; Lee et al., 1997); *Ascl2* encodes a placenta-specific transcription factor (Guillemot et al., 1995; McLaughlin et al., 1996); and *Phlda2* regulates placental growth and hormone synthesis (Jensen et al., 2014).

The *Cdkn1c/Kcnq1ot1* locus is primarily regulated by the lncRNA *Kcnq1ot1*. The *Kcnq1ot1* promoter resides within KvDMR1, which is methylated on the maternal allele and unmethylated on the paternal allele. *Kcnq1ot1* transcription is essential for proper imprinting of the *Cdkn1c/Kcnq1ot1* locus. The unmethylated paternal KvDMR1 promotes the transcription of *Kcnq1ot1* to silence the linked imprinted genes, likely via the recruitment of repressive epigenetic modulators including histone modifiers and DNA methyltransferases (Pandey et al., 2008; Mohammad et al., 2010). Conversely, the maternal KvDMR1 methylation silences *Kcnq1ot1*, enabling the expression of the linked imprinted genes. This mechanism is supported by numerous studies in mouse. For example, deleting KvDMR1 on the paternal allele disrupted allele-specific regulation of *Cdkn1c* and other linked genes [Fig. 2C, 2.8kb-KvDMR1Δ (Fitzpatrick et al., 2002) and 3.6kb-DMRΔ (Mancini-DiNardo et al., 2006)]. Deletion of the paternal *Kcnq1ot1* promoter in mice led to abnormal methylation and expression of genes in the cluster [Fig. 2C, 244bp-PromΔ (Mancini-DiNardo et al., 2006) and 564bp-Kcnq1ot1^PO^ (Schultz et al., 2015)]. Indeed, the transcriptional activity of the *Kcnq1ot1* promoter ensures KvDMR1 hypomethylation and repression of *Cdkn1c*. Interestingly, others have shown that *Kcnq1ot1* may control the locus in most, but not all, tissues. Truncation of the paternal *Kcnq1ot1* transcript de-repressed paternal *Cdkn1c* expression in brain, heart, gut and placenta, but not in liver, kidney and lung [Fig. 2C, pA/YJ69 (Shin et al., 2008)]. Thus, although the full *Kcnq1ot1* transcript is required to silence *Cdkn1c* and other genes in the cluster in most tissues, an alternative mechanism, independent of *Kcnq1ot1*, may mediate *Cdkn1c* silencing in some tissues.

## Genetic and epigenetic errors of BWS

Most BWS patients have genetic or epigenetic errors at the *H19/IGF2* and/or the *CDKN1C/KCNQ1OT1* clusters, such as DNA methylation perturbations, copy-number variants and loss-of-function mutations of imprinted genes (Table 1). Indeed, the various epigenotypes of BWS patients correlate with different phenotypes. For example, hemihypertrophy and hypoglycemia are associated with paternal uniparental disomy (pUPD) of 11p15 (DeBaun et al., 2002). Individuals with abnormal IC1 methylation are reported to have increased tumor risk (Bliek, 2001). *H19*/*IGF2* imprinting is consistently dysregulated in BWS patients presenting with Wilms' tumors (Dao et al., 1999; Frevel et al., 1999). Gain of methylation (GoM) on IC1 is strongly correlated with neonatal macrosomia and disproportionate overgrowth (Mussa et al., 2016). Premature birth is associated with loss of methylation (LoM) on IC2 or *CDKN1C* mutations (Mussa et al., 2016). Finally, patients with abdominal wall defects exhibit a higher frequency of abnormal methylation on the *CDKN1C/KCNQ1OT1* cluster (DeBaun et al., 2002). Thus, elucidating the epigenetic mechanisms for the BWS imprinted clusters would increase our understanding of how certain mutations cause defined pathological phenotypes, ultimately leading to better treatment plans for patients. Given the conserved biology and regulation of IC1/IC2, the investigation of dysregulated human imprinting using mouse models has been particularly informative.

**Table 1.**
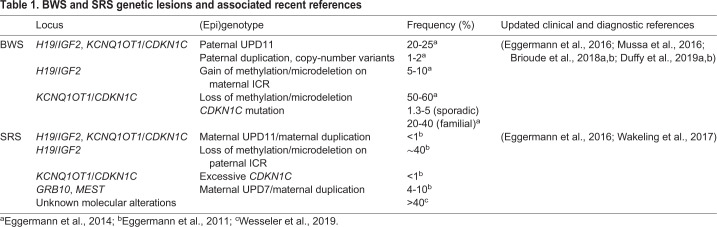
**BWS and SRS genetic lesions and associated recent references**

## pUPD11 in BWS

Twenty to 25% of BWS patients have pUPD of chromosome 11p15.5, where both copies of 11p15.5 are inherited from the father (Fig. 1D and Table 1). Modeling pUPD11 in mice has been difficult because pUPD of the corresponding region in mice (distal chromosome 7) is lethal at midgestation (McLaughlin et al., 1996; Rentsendorj et al., 2010; Singh et al., 2011). Mouse embryos with pUPD7 were developmentally delayed at 10.5 days post coitum (dpc), lacked placental spongiotrophoblasts and were resorbed before 13.5 dpc. Although these embryos lacked *H19* and exhibited increased *Igf2* expression, deleting paternal *Igf2* failed to rescue their viability (Rentsendorj et al., 2010). Similarly, when only the paternal *Cdkn1c/Kcnq1ot1* cluster is left functional due to the truncation of maternal distal chromosome 7, lethality was observed by 10.5 dpc in mice [DelTel7 (Oh et al., 2008)]. The difference in UPD lethality between human and mouse may arise from the typical mosaic presentation of UPD and non-UPD cells in human patients, whereas all cells in pUPD7 mice are constitutively UPD. Nevertheless, and perhaps more importantly, midgestation lethality in mouse may be caused by loss of expression of the maternally expressed imprinted gene *Ascl2*, which is not imprinted in humans (Fig. 2A) (Westerman et al., 2001; Miyamoto et al., 2002; Rentsendorj et al., 2010). Consistent with this hypothesis, the embryonic lethality of the aforementioned DelTel7 mouse model was partially rescued by restoring *Ascl2* expression (Tunster et al., 2018).

## The *H19/IGF2* cluster in BWS

Five to 10% of BWS patients exhibit GoM or IC1 hypermethylation at the *H19/IGF2* locus (Table 1). DNA methylation antagonizes CTCF binding (Kanduri et al., 2000; Wang et al., 2012). Therefore, hypermethylation of IC1 on the normally unmethylated maternal allele can prevent CTCF binding, which disfavors *H19* expression and increases *IGF2* expression. CTCF is indispensable for both insulating *IGF2* and maintaining the unmethylated state of maternal IC1. In mice, targeted deletion of all four CTCF binding sites (CTSs) within the maternal *H19/Igf2* ICR resulted in hypermethylation of mutant maternal ICR, loss of CTCF-mediated insulator function and therefore biallelic *Igf2* expression [Fig. 1C, DMD-ΔR (Engel et al., 2006)]. Similar results were obtained when the four CTSs were mutated on the maternal *H19*/*Igf2* ICR, where the mice exhibited variable acquisition of DNA methylation and maternal *Igf2* activation [Fig. 1C, CTS mutation (Schoenherr et al., 2003; Szabo et al., 2004)]. These models underscore the importance of proper CTCF binding to the ICR for allele-specific regulation of *H19/Igf2* expression.

Given that *H19* and *IGF2* are key regulators in development, it is expected that perturbations in gene dose promote abnormal growth. For example, overexpression of *Igf2* caused by introducing an *Igf2* transgene in mice causes fetal overgrowth similar to the human BWS phenotype (Sun et al., 1997). A mouse carrying an *H19* deletion on the maternal allele exhibited fetal overgrowth and tissue-specific activation of maternal *Igf2* [Fig. 1C, *H19*Δ3 (Ripoche et al., 1997) and loxΔ*H19* (Schmidt et al., 1999)]. Additionally, *H19* and *IGF2* can work together to control growth. Early work suggested that *H19* is a trans-acting repressor of *Igf2* (Li et al., 1998). Transgenic overexpression of *H19* in *H19*Δ3 mice restored *H19* and *Igf2* expression to wild-type levels and rescued the overgrowth phenotype (Gabory et al., 2009; Martinet et al., 2016), suggesting that *H19* may function as a repressor of *Igf2.* In conclusion, absence of *H19* and/or increased *Igf2* can lead to overgrowth in early development.

The causes of IC1 hypermethylation remain unclear. The relationship between IC1 mutations, IC1 hypermethylation and BWS phenotypes is complex (Table 1) (Brioude et al., 2018a,b; Duffy et al., 2019b). IC1 hypermethylation is often associated with microdeletions (Sparago et al., 2004), which may change the spacing of CTSs and affect CTCF binding, insulator activity and DNA methylation of IC1. Therefore, it is possible that the variable BWS phenotypes may stem from the spatial arrangement of CTCF binding sites specific to each microdeletion (Beygo et al., 2013). For example, in one representative BWS family, transmission of a specific IC1 mutation over generations was accompanied by a gradual increase in DNA methylation (Berland et al., 2013). Conversely, another BWS family with an IC1 microdeletion had no IC1 methylation abnormalities (Prawitt et al., 2005). In this study, a family member carrying a maternally inherited IC1 microdeletion and a duplication of chromosome 11p15 presented with BWS symptoms, whereas other relatives with the same maternal IC1 deletion did not. Additional studies document incomplete penetrance of BWS features despite all relatives carrying the same IC1 microdeletion (Cerrato et al., 2008; Berland et al., 2013). One factor that may contribute to this clinical heterogeneity is the developmental stage at which the IC1 deletion occurs. For example, one BWS study identified a point mutation within IC1 that originated from the paternal allele of a patient's grandmother (Berland et al., 2013). During oogenesis in this grandmother, IC1 reprogramming (i.e. removal of paternally methylated epigenetic marks and establishment of maternally unmethylated marks) could have been disrupted by the mutation, resulting in incomplete demethylation of the mutant IC1. Once this hypermethylated IC1 was maternally inherited, methylation gradually accumulated on the mutant IC1 over generations. Consistently, mouse studies showed imprinted *H19* expression was not affected when the paternal *H19/Igf2* ICR was deleted after methylation of the *H19* promoter was established [Fig. 1C, DMR^Δ^ (Srivastava et al., 2000)].

It is important to note that genetic lesions at IC1 are not always accompanied by hypermethylation, and the aberrant methylation patterns are not necessarily caused by IC1 mutations. For example, a subset of BWS patients exhibited IC1 hypermethylation but did not carry any identified genetic lesions (Cerrato et al., 2008). Another factor that can explain the heterogeneity in BWS is epigenetic mosaicism (Abi Habib et al., 2017). IC1 hypermethylation is often mosaic in BWS patients and is independent of the mutations (Cerrato et al., 2008). Mosaic expression was also observed at a single-cell level in mice harboring ICR mutations (Ginart et al., 2016). The fact that hypermethylation exhibits a mosaic pattern suggests that the hypermethylation is acquired after fertilization instead of in the germline.

Two important questions remain regarding hypermethylation and IC1 microdeletions associated with BWS: (1) do both maternally derived genetic lesions and hypermethylation cause BWS, or (2) is GoM the consequence of the microdeletion? To understand the relationship between microdeletions and IC1 hypermethylation, researchers have utilized mouse models with partial deletions in the *H19*/*Igf2* ICR. Although the regulatory mechanisms of the locus are well conserved, the mouse *H19*/*Igf2* ICR differs from the human IC1 in the number of CTSs, genomic size and nucleotide sequence (Fig. 1A,B). Consequently, the optimal way to understand BWS lesions using a mouse model is through ICR deletions that remove CTCF binding sites. Deletion of two of the four CTSs on the maternal *H19/Igf2* ICR resulted in tissue-specific activation of maternal *Igf2* and increased birth weight, despite the normal methylation of the mutant ICR [Fig. 1C, Δ2,3 (Ideraabdullah et al., 2014)]. This model demonstrates that partial deletion of the *H19/Igf2* ICR is not always accompanied by ICR hypermethylation and that ICR hypermethylation is not strictly required for abnormal *H19*/*Igf2* expression and growth. In contrast, when a larger ICR deletion containing three CTSs was maternally transmitted, the mutant maternal ICR gained methylation and maternal *Igf2* was activated [Fig. 1C, ΔDMD (Thorvaldsen et al., 1998, 2002, 2006)]. Similarly, when all four CTSs were deleted or mutated in the maternal *H19/Igf2* ICR, the mutant ICR acquired methylation and *H19/Igf2* imprinting was disrupted ubiquitously [Fig. 1C, CTS mutation (Schoenherr et al., 2003; Szabo et al., 2004; Engel et al., 2006)]. These models suggest that the nature of ICR deletions determines whether there will be accompanying hypermethylation, although it is unclear whether the deletion size, position or sequence is critical to determine whether the ICR is protected from ectopic hypermethylation. Therefore, a subset of IC1 microdeletions that disrupt the key aspects of IC1 function may result in its hypermethylation and, ultimately, BWS.

As stated above, due to a lack of ICR sequence conservation, mice are limited in modeling the exact BWS mutations observed in humans. To recapitulate human mutations more precisely, Hur and colleagues generated a mouse strain with human IC1 (hIC1) sequence substituting the endogenous murine *H19*/*Igf2* ICR [Fig. 1A, hIC1 (Hur et al., 2016)]. The paternally transmitted hIC1 allele failed to acquire and maintain the normal hypermethylation (see further discussion below). However, when maternally inherited, the hIC1 allele exhibited normal hypomethylation and CTCF insulator function, suggesting a possible system to model human BWS mutations endogenously in mice. For example, Freschi et al. designed a mouse strain with hIC1 including a 2.2-kb deletion found in BWS patients [Fig. 1A, hIC1Δ2.2 (Freschi et al., 2018)]. Maternal transmission of this mutant hIC1 resulted in tissue-specific overexpression of *Igf2*, successfully mimicking clinical BWS phenotypes including pre-/postnatal overgrowth, nephromegaly, macroglossia and kidney asymmetry. Notably, mutant hIC1 gained methylation when maternally transmitted, possibly because the removal of three CTSs reduced CTCF binding to the IC1. Thus, humanized mouse models offer a powerful tool to mimic the epigenetic and genetic errors found in human BWS patients and examine their physiological consequences.

## The *CDKN1C/KCNQ1OT1* cluster in BWS

Fifty percent of BWS patients exhibit LoM at IC2 (Table 1). In these cases, the lncRNA *KCNQ1OT1* is predicted to be ectopically expressed from the maternal allele, which silences the growth suppressor *CDKN1C* and other maternally expressed genes. Importantly, loss of *CDKN1C* expression is a major cause of the overgrowth phenotype in BWS patients, and knockout of maternal *Cdkn1c* in mice causes similar fetal overgrowth, as well as cleft palate, abdominal wall defects and placental defects such as placentomegaly that approximate the BWS phenotype (Yan et al., 1997; Zhang et al., 1997; Tunster et al., 2011).

Dysregulation of other IC2-controlled genes in addition to *CDKN1C* can contribute to BWS-like phenotypes. One such pathway involves *PHLDA2*, a maternally expressed gene within the IC2 cluster (Fig. 2). In mice, maternal inheritance of a *Phlda2* deletion resulted in placentomegaly (Frank et al., 2002; Salas et al., 2004), which suggests that the dysregulation of *Phlda2* expression caused by disrupted imprinting of the IC2 cluster can lead to adverse placental phenotypes, one of the hallmark BWS symptoms.

**Fig. 3.**
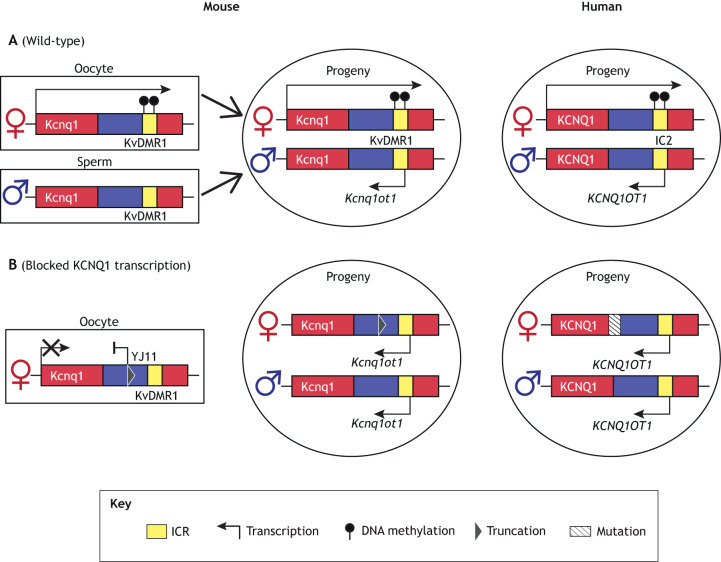
**Establishment of DNA methylation at the *CDKN1C/KCNQ1OT1* imprinted locus.** (A) The transcription of *Kcnq1* in mouse oocytes is postulated to establish maternal-allele-specific methylation on KvDMR1, which silences maternal *Kcnq1ot1* expression in the progeny. The allele-specific regulation of this cluster is conserved in humans. (B) Truncating the maternal *Kcnq1* transcript in mice [Ap/YJ11 (Singh et al., 2017)] resulted in hypomethylation on the maternal KvDMR1. In humans, maternally inherited mutations, which disrupt *KCNQ1* transcription, are hypothesized to disrupt the establishment of methylation on maternal IC2, which can lead to LoM on IC2 in the progeny (Niemitz et al., 2004; Beygo et al., 2019; Valente et al., 2019). See text for details.

In a subset of BWS patients, LoM on IC2 may result from maternally transmitted mutations, particularly those that affect transcription. Notably, work in mice has shown that establishment of maternal-allele-specific methylation on KvDMR1 requires active transcription of *Kcnq1* through KvDMR1 during oocyte maturation (Fig. 3A) (Chotalia et al., 2009; Singh et al., 2017). Thus, a defect in transcription may result in the failure to establish proper methylation on maternal KvDMR1 (Fig. 3B). Human BWS cases support this model. Valente et al. described a BWS patient with a maternally inherited mutation affecting *KCNQ1* transcription and complete LoM at IC2 (Valente et al., 2019). In a second BWS family, disruption of *KCNQ1* transcription by translocation of *KCNQ1* exons to a different chromosome was associated with abnormal IC2 methylation (Beygo et al., 2019). In a third report, a maternally inherited microdeletion in *KCNQ1OT1* coincided with IC2 LoM, *CDKN1C* silencing and BWS features (Niemitz et al., 2004). To genetically dissect the role of transcription in IC2 imprinting in more detail, mouse models harboring a truncated maternal *Kcnq1* transcript just prior to KvDMR1 have been generated. These mice exhibited LoM on the maternal KvDMR1, biallelic expression of *Kcnq1ot1* and repression of normally maternally expressed genes, including *Cdkn1c* [Figs 2C and 3B, Ap/YJ11 (Singh et al., 2017)]. Together, these human and mouse studies suggest that a mutation disrupting *Kcnq1* transcription leads to the loss of imprinting of the entire cluster.

Despite solid evidence for this maternally based transcription model for LoM, the origin of IC2 hypomethylation for many BWS patients remains unclear. Given the typical mosaic pattern of LoM in different tissues, it is likely that the methylation defect occurs after fertilization. This is particularly relevant for patients conceived using assisted reproductive technologies (ART) and, more specifically, *in vitro* fertilization (IVF). IVF is associated with a higher-than-expected frequency of BWS and SRS cases that involve DNA methylation defects (Johnson et al., 2018). One possible explanation for this observation is that IVF involves manipulations of gametes and embryos at a time of epigenetic reprogramming. The genome undergoes an extensive DNA methylation reprogramming after fertilization, but DNA methylation at ICRs must survive this reprogramming (Morgan et al., 2005). The environmental perturbations inherent to ART, i.e. hormone treatments and *ex vivo* manipulations, may affect the fidelity of DNA methylation at imprinted genes in the early embryo (Vrooman and Bartolomei, 2017). Consistent with this hypothesis, mouse models of IVF have shown that simply culturing embryos *ex vivo* can result in significant changes in DNA methylation (Doherty et al., 2000; Mann et al., 2004). Therefore, environmental insults during early pregnancy may cause BWS in children without inherited genetic mutations.

## *CDKN1C* mutations and loss of expression

Five to 10% of all BWS patients carry deficiency of or loss-of-function mutations in the growth suppressor *CDKN1C* (Table 1) (Choufani et al., 2013; Mussa et al., 2016). Early clinical work determined that maternal *CDKN1C* expression was absent in Wilms' tumors (Matsuoka et al., 1996), suggesting that maternally inherited loss-of-function mutations in *CDKN1C* can cause some of the classic symptoms of BWS. This human mutation inspired the generation of mouse models with *Cdkn1c* mutations to study its role in BWS (Yan et al., 1997; Zhang et al., 1997; Tunster et al., 2011). Yan et al. reported an increase in the number of apoptotic cells in affected organs, underscoring the role of *Cdkn1c* as a cell cycle regulator (Yan et al., 1997). *Cdkn1c* mutant embryos were oversized at midgestation (Tunster et al., 2011) and showed abnormalities in muscles covering the abdominal wall, recapitulating the abdominal wall defects seen in BWS patients (Zhang et al., 1997; Tunster et al., 2011). To note, the fetal overgrowth of *Cdkn1c* mutant mice was reversed rapidly in late gestation. Tunster et al. have suggested that intrauterine competition in mice may have masked the fetal overgrowth caused by the lack of *Cdkn1c*, and singleton pregnancy in human allows presentation of the overgrowth phenotype in BWS offspring (Tunster et al., 2011).

## SRS

SRS (OMIM #180860) is a fetal undergrowth genetic disorder with an incidence range of 1 in 30,000 to 100,000 (Wakeling et al., 2017). Representative SRS symptoms include lower birth weight, growth restriction lasting to adulthood, skeletal/limb asymmetry, cognitive impairment together with delayed language development, macrocephaly, fifth-finger clinodactyly and characteristic triangular face (Wakeling et al., 2017). SRS is a ‘sister’ epigenetic disorder of BWS. The dysregulation of the same gene cluster can cause either the overgrowth observed in BWS or undergrowth in SRS. Consistently, depending on the parental origin, the same genetic defect in imprinted clusters can result in increased or decreased expression of these imprinted growth regulators. Here, we discuss how both human and mouse studies have informed our understanding of the abnormal imprinting patterns of IC1 and SRS.

## The *H19/IGF2* cluster in SRS

Loss of IC1 methylation, independent of a genetic mutation *in cis*, is found in 50% of SRS patients (Table 1). Normally, methylation on paternal IC1 prevents CTCF binding and silences paternal *H19*. In SRS, the hypomethylated paternal IC1 is proposed to enable the formation of an ectopic CTCF insulator, as demonstrated in an SRS mouse model (Engel et al., 2004; Gicquel et al., 2005). As a result, the paternal *H19* is activated and *IGF2* expression is decreased. Altered *H19* and/or *IGF2* expression can lead to abnormal growth because *H19* and *IGF2* are growth regulators. In mice, paternal-specific deletion of *Igf2* resulted in total loss of *Igf2* expression and pre- and postnatal growth restriction (DeChiara et al., 1991; Haley et al., 2012). Moreover, transgenic *H19* overexpression caused embryonic growth restriction (Gabory et al., 2009). Therefore, improper *H19/Igf2* expression caused by IC1 LoM can cause abnormal development in SRS.

The consequences of IC1 LoM found in humans are explored further in the aforementioned humanized mouse model [Fig. 1A, hIC1 (Hur et al., 2016)]. In these mice, the paternally transmitted hIC1 failed to establish proper methylation during spermatogenesis, enabling CTCF to function as an insulator on the paternal allele. Therefore, the offspring of these sires exhibit highly elevated *H19* expression, undetectable *Igf2* expression, severe growth restriction and perinatal lethality. In a different mouse model, mutating CpGs within the CTSs of the paternal *H19/Igf2* ICR caused LoM [Fig. 1C, DMD-9CG (Engel et al., 2004)]. This mutant ICR was unable to maintain methylation after fertilization, thereby promoting CTCF binding and ectopic insulator function on the paternal allele. These offspring also display activated paternal *H19* and reduced *Igf2* expression, culminating in restricted embryonic growth.

What can cause the IC1 LoM in SRS patients? As with BWS methylation abnormalities, SRS patients typically present with a mosaic pattern of methylation (Table 1) (Eggermann et al., 2016). This mosaic pattern suggests that LoM may occur postfertilization and may be linked to an unfavorable embryonic/fetal environment. ART/IVF strategies represent an example of an environmental insult on the methylome, and, similar to BWS, SRS appears in IVF conceptuses more often than predicted by random chance (Hattori et al., 2019). Additionally, ART-conceived SRS patients have higher variability in methylation compared to spontaneously conceived SRS individuals (Hattori et al., 2019). Because ART procedures occur in the developmental window when the epigenome is reprogrammed, the epigenetic regulators responsible for maintaining imprints at IC1 may be perturbed, predisposing ART babies to a higher risk for SRS.

IC1 LoM can occasionally be caused by genetic lesions. SRS patients with IC1 hypomethylation may also present with paternally inherited IC1 deletions (Abi Habib et al., 2017). A BWS-related IC1 deletion that caused GoM on maternally inherited IC1 in a different family member caused LoM on IC1 when paternally inherited (Kraft et al., 2019). As described in the hIC1Δ2.2 mouse model, paternal transmission of the mutant hIC1 allele resulted in incomplete methylation of the paternal IC1, recapitulating LoM in SRS [Fig. 1A, hIC1Δ2.2 (Freschi et al., 2018)]. The incomplete establishment of methylation on the mutant IC1 may be due to the inability of the mutated allele to be recognized by DNA methyltransferases during spermatogenesis and/or the subsequent failure to maintain the previously established methyl marks. Animals with a hypomethylated paternal ICR showed high *H19* and low *Igf2* expression, and were pre- and postnatally growth restricted. The liver and kidneys were more severely affected than other organs in this model, closely resembling the organ-specific growth restriction in SRS.

The KRAB zinc finger protein ZFP57 is another factor that can affect DNA methylation of the *H19/IGF2* locus. ZFP57, which interacts with ICRs in a methylation-dependent manner, binds to methylated CpGs in ICRs and protects them from genome-wide demethylation during the postfertilization reprogramming window (Li et al., 2008; Strogantsev et al., 2015). This binding enables the preservation of parental allele-specific methylation of imprinted clusters. Previous work suggested that ZFP57 was involved in the etiology of IC1 hypomethylation in SRS (Hirasawa and Feil, 2008), in that loss-of-function mutation of *ZFP57* resulted in mosaic hypomethylation of many imprinted loci (Li et al., 2008; Mackay et al., 2008). Consistently, Sparago et al. suggested that, in the hIC1 transgenic mouse model (Hur et al., 2016), the paternally transmitted hIC1 could not be properly methylated possibly because the inserted hIC1 sequence lacked a ZFP57 binding site. They suggested that insertion of an additional ZFP57 binding site in the hIC1 construct would enable ZFP57 to bind and protect paternally established methylation (Sparago et al., 2018). However, patients with transient neonatal diabetes mellitus 1 caused by loss-of-function mutations of *ZFP57* exhibited normal methylation of IC1 (Mackay et al., 2008; Boonen et al., 2013). Moreover, SRS patients with IC1 hypomethylation were found to have no functional mutation of ZFP57 (Spengler et al., 2009). Although the role of ZFP57 at IC1 remains to be determined, it is likely that other zinc finger proteins, such as the recently described ZFP445, preserve DNA methylation and may be mutated in SRS patients (Takahashi et al., 2019).

Mutations in the *H19/IGF2* cluster other than IC1 LoM are also found in SRS patients. Chromosomal structural variations in the *H19/IGF2* enhancer region were reported in a group of SRS patients (Grønskov et al., 2011). Paternally inherited genetic lesions that disrupt the interaction between the mesodermal enhancer and the *IGF2* promoter resulted in delayed growth in patients. In mice, deletion of the endodermal *H19/Igf2* enhancers on the paternal allele led to growth-restricted offspring (to 70% of that of wild type at birth) and reduced *Igf2* expression in endodermal tissues such as liver and kidney [Fig. 1C, EndoEnhΔ (Leighton et al., 1995)]. Additionally, paternally transmitted disruption of mesodermal *H19/Igf2* enhancers resulted in growth retardation (50% of wild type at birth), repressing *Igf2* expression in mesodermal tissues including tongue and kidney [Fig. 1C, Mnt (Davies and Reik, 2002)]. The deletion of mesodermal *H19/Igf2* enhancers on the paternal allele also resulted in reduced *Igf2* expression in mesodermal tissues [Fig. 1C, VM3 (Kaffer et al., 2001)].

## mUPD11 and duplication of maternal chromosome 11p15

Rarely, SRS patients have maternal UPD (mUPD) or maternal duplication of chromosome 11p15 [Table 1, Fig. 1D (Luk et al., 2016)]. In mice, mUPD of mouse distal chromosome 7, which includes the *H19/Igf2* and *Cdkn1c/Kcnq1ot1* clusters, resulted in growth deficiency and perinatal lethality (Han et al., 2010). In the presence of two maternal distal chromosome 7 alleles, the maternally expressed genes are overexpressed and the paternally expressed genes are silent. Accordingly, mUPD7 mice had loss of *Igf2* and increased *H19* and *Cdkn1c* expression, which would result in severe undergrowth. To understand the source of the perinatal lethality, the *H19/Igf2* ICR and the *Cdkn1c* gene were deleted in mUPD7 mice. When *H19/Igf2* ICR was deleted on one allele, the mUPD7 pups were viable, but the growth restriction persisted, albeit to a smaller extent. Here, *Igf2* expression was restored to wild-type levels, but *Cdkn1c* expression remained high. Deletion of *Cdkn1c* from the paternal allele of mUPD7 mice restored embryonic weight to the wild-type level but lethality persisted. This demonstrates that excessive *Cdkn1c* expression was not responsible for mUPD7 lethality. Indeed, IC1 and IC2 clusters may contribute uniquely to the growth dysregulation in mUPD11 SRS patients.

## mUPD7 and duplication of chromosome 7

Five to 10% of SRS patients are reported to have duplication (Monk et al., 2000; 2002; Eggermann et al., 2012b) or mUPD on chromosome 7 (Yuan et al., 2016), where growth factor receptor-bound protein 10 [*GRB10*; also known as maternally expressed gene 1 (*MEG1*)] resides. SRS patients with mUPD7 are more likely to have verbal dyspraxia, learning difficulties, myoclonus dystonia and autistic spectrum disorder compared to other SRS subgroups (Wakeling et al., 2017). *GRB10* functions as a growth suppressor, binding to insulin and IGF1 receptors to inhibit the growth-promoting activity of IGF. Therefore, increased *GRB10* in SRS patients can result in an undergrowth phenotype. *Grb10* is located on chromosome 11 in mouse, and the somatic isoform is maternally expressed. Maternal duplication of mouse proximal chromosome 11, which includes the *Grb10* locus, resulted in prenatal growth retardation (Miyoshi et al., 1998). In contrast, paternal duplication of the same region resulted in growth enhancement, proving that the allele-specific regulation of *Grb10* expression is crucial for normal development. To note, *Grb10* isoforms are expressed from different parental alleles in somatic and neuronal tissues in mice. Absence of the paternal, brain-specific isoform of *GRB10* might thus cause the brain-specific phenotypes in mUPD7 patients.

Another imprinted gene implicated in SRS is located on chromosome 7q: mesoderm-specific transcript [*MEST*; also known as paternally expressed gene 1 (*PEG1*)]. The *MEST* promoter is methylated on the maternal allele, and *MEST* is expressed from the paternal allele (Riesewijk et al., 1997). Several SRS patients with mild symptoms were reported to have mUPD for chromosome 7q (Hannula et al., 2001; Reboul et al., 2006; Eggermann et al., 2008) or a deletion on paternal 7q spanning *MEST* (Eggermann et al., 2012a). In mice, loss of *Mest* expression from the paternal allele resulted in pre- and postnatal growth restriction (Lefebvre et al., 1998), suggesting a growth regulatory role for *MEST*.

## Mutations related to the *CDKN1C/KCNQ1OT1* cluster

A small number of SRS patients have mutations in the *CDKN1C/KCNQ1OT1* cluster. Maternally inherited activating mutations of *CDKN1C* were found in SRS patients (Table 1; Brioude et al., 2013). A microduplication encompassing the *CDKN1C/KCNQ1OT1* cluster, but not the *H19/IGF2* cluster, was found on the maternal allele of an SRS patient (Schönherr et al., 2006). In a different SRS family, a microduplication of the *CDKN1C/KCNQ1OT1* cluster resulted in SRS only when maternally inherited (Bonaldi et al., 2011). Transgene modeling this duplicated region led to increased *Cdkn1c* expression in mice, together with growth reduction, which was dependent on *Cdkn1c* dosage and lasted to adulthood (Andrews et al., 2007). This mouse model also showed neonatal hypoglycemia, decreased body fat in adults (Van De Pette et al., 2016) and behavioral abnormalities (McNamara et al., 2016), phenotypes that recapitulate representative SRS features. Mice with deletion of paternal KvDMR1 exhibited biallelic expression of *Cdkn1c* and fetal growth retardation, further demonstrating the growth repressor functions of *Cdkn1c* [Fig. 2C, 2.8kb-KvDMR1Δ (Fitzpatrick et al., 2002) and 3.6kb-DMRΔ (Mancini-DiNardo et al., 2006)]. When the paternal *Cdkn1c* was activated due to the truncation of the paternal *Kcnq1ot1* transcript, mice exhibited growth deficiency [Fig. 2C, pA/YJ69 (Shin et al., 2008)]. Transgenic overexpression of *Phlda2* and *Slc22a18* resulted in placental growth retardation without involving a change in *Cdkn1c* expression (Salas et al., 2004; Tunster et al., 2010). Further research showed that the increased *Phlda2* expression contributed more to placental insufficiency and asymmetric growth restriction (Tunster et al., 2014). These results suggest that other genes in the cluster can contribute the growth restriction phenotype of SRS.

## MLID: lesions on multiple loci

Multi-locus imprinting disturbances (MLID) exhibit imprinting defects at multiple loci, in addition to the locus primarily relevant to the patient's symptoms (Sanchez-Delgado et al., 2016). Up to 25% of BWS patients with IC2 LoM and 10% of SRS patients with IC1 LoM were reported to have MLID (Azzi et al., 2009; Eggermann et al., 2011). Although the exact cause of MLID is unknown, some MLID patients carry defects in *trans* regulators such as ZFP57 (Mackay et al., 2008), NLRP2 (Meyer et al., 2009), NLRP5 (Docherty et al., 2015) and PADI6 (Begemann et al., 2018) that may contribute to the variable phenotypes. Among these, deletion of the oocyte-specific gene *Nlrp5* (also known as *Mater*) caused sterility in female mice, producing embryos that developmentally arrest at the two-cell stage (Tong et al., 2000). *Nlrp2-*deficient females were subfertile and produced developmentally delayed and smaller offspring (Mahadevan et al., 2017), with more rapidly decreasing reproductive rates, as one would expect with accelerated aging (Kuchmiy et al., 2016). Mice do not have an ortholog of *NLRP7* and, therefore, defects in *NLRP7* cannot be modeled. Overall, it would be important to understand the roles of these *trans* regulators that may be crucial for establishing and maintaining DNA methylation on multiple imprinted loci.

## Conclusions and future directions

Mouse models have been widely utilized to understand how genomic imprinting is established, maintained and inherited in humans. To mimic the pathologic genotypes of imprinting disorders such as BWS and SRS in humans, researchers have generated numerous mouse models. These mouse models revealed (1) the main regulatory mechanisms of imprinted clusters such as *H19/Igf2* and *Cdkn1c/Kcnq1ot1*, (2) how the lack of key components of imprinted clusters lead to dysregulation, and (3) the roles of linked genes in the imprinted clusters.

Although manipulating mouse genomes has provided important knowledge, as discussed in this Review, modeling human epigenetic mutations in mice has several limitations. For example, pUPD11 is a significant cause of BWS, but modeling pUPD11 in mice has been challenging due to embryonic lethality. This prenatal death is caused by loss of expression of the imprinted *Ascl2* gene, which is not imprinted in humans (Westerman et al., 2001; Miyamoto et al., 2002; Rentsendorj et al., 2010). Additionally, the mosaicism observed in human pUPD11 patients is not evident in mice. Together, these factors complicate the modeling of human pUPD11.

To overcome such limitations, several other methods have been developed to study imprinting disorders, including human induced pluripotent cells (hiPSCs). Derived from BWS patients' fibroblasts, the strength of this system is the preserved pathological genetic environment. This eliminates the need to manipulate the genome to achieve the desired mutations and therefore avoids the possible off-target effects of gene editing. Another advantage of the system is that hiPSCs can be differentiated into clinically relevant tissues that are difficult to study in human BWS patients. Combining information gleaned from hiPSCs and traditional mouse models will enhance our understanding of the genetic mechanisms underlying imprinting disorders such as BWS and SRS.
